# Emerging trends and hotspots in cognitive behavioral therapy for chronic pain: a bibliometric analysis

**DOI:** 10.3389/fmed.2026.1788247

**Published:** 2026-06-17

**Authors:** Qiuyu Jin, Yukun Wen, Lili Wu, Zijian Wang, Yang Zhang

**Affiliations:** 1 First Affiliated Hospital of Heilongjiang University of Chinese Medicine, Harbin, China; 2 Guangxi University of Chinese Medicine, Nanning, China; 3 Shenzhen Hospital of Guangzhou University of Chinese Medicine (Futian), Shenzhen, China

**Keywords:** bibliometric, chronic pain, cognitive behavioral therapy, hotspots, research fronts

## Abstract

**Background:**

Despite growing interest in Cognitive Behavioral Therapy (CBT) for chronic pain, bibliometric analyses in this field remain scarce. This study provides a multi-dimensional bibliometric analysis of literature from 2005 to 2024 to summarize research trends and guide future directions.

**Methods:**

Literature was retrieved from the Web of Science Core Collection and clinical trial records from PubMed (2005–2024). Data analysis and visualization were conducted using Microsoft Excel, Scimago Graphica, VOSviewer, CiteSpace, and the R-package “bibliometrix” to evaluate publication trends, institutional contributions, and clinical design characteristics.

**Results:**

A total of 1,070 papers involving 67 countries, 1,584 institutions, and 4,820 authors were identified. The United States dominated the field, contributing 59.16% of total publications and ranking first in citations. The University of Washington emerged as the most productive institution, while The Journal of Pain led in total publications (7.01%). Analysis of keywords—such as Depression, Sleep, Anxiety, and Quality of Life—highlights current hotspots. Research increasingly focuses on validating digital interventions, managing comorbidities (e.g., insomnia, mood disorders), and tailoring personalized treatment strategies for specific populations.

**Conclusion:**

Research on CBT for chronic pain is flourishing. This first systematic bibliometric analysis identifies digital interventions, comorbidity management, and personalized treatment as primary research hotspots. It provides a strategic roadmap for future mechanism-oriented clinical trials, interdisciplinary integration, and real-world applications.

## Introduction

Chronic pain is defined as pain that persists for more than 3 months, typically exceeding the expected duration of normal healing (1). In contemporary pain science, chronic pain is categorized into three distinct mechanistic phenotypes: nociceptive pain, arising from actual or threatened damage to non-neural tissue; neuropathic pain, caused by a lesion or disease of the somatosensory nervous system; and nociplastic pain, characterized by altered nociception despite no clear evidence of actual or threatened tissue damage or somatosensory pathology (2–4). Chronic pain not only affects physical health but can also lead to emotional problems such as anxiety and depression, significantly impacting patients’ quality of life. It is estimated that 20.5% of adults suffer from chronic pain, a condition influenced by biological, psychological, and social factors, requiring management that addresses not only its biological causes but also its psychological and social impacts and consequences (5). Psychosocial factors play a significant role in pain and related physical and psychosocial dysfunction. Cognitive Behavioral Therapy (CBT) is a psychotherapeutic approach designed to improve mood and behavior by changing an individual’s thought patterns and behaviors (6). CBT is particularly suitable for patients with chronic pain because it helps them identify and adjust negative cognitions about pain, thereby enhancing coping abilities (7). Studies have shown that CBT can effectively reduce chronic pain and its associated psychological distress, improving patients’ overall quality of life (8). Over the past 60 years, parallel advancements in the scientific understanding of pain and the development of cognitive and behavioral therapies have led to the widespread application of CBT in addressing chronic pain issues (9–11). In fact, CBT is now a mainstream treatment method, whether used alone or in combination with medical or interdisciplinary rehabilitation treatments, for all types of chronic pain patients (6).

Although existing studies have explored the role of CBT in chronic pain, the understanding of its essence remains in the preliminary stages (12). Furthermore, the rapid growth in the number of publications may make it difficult for researchers to fully grasp the key advancements and future directions of CBT in the field of chronic pain (13). Therefore, a systematic analysis of the hotspots and trends in this specific area is particularly necessary. Bibliometrics, as an emerging knowledge synthesis method, can explore significant development trends in research fields by identifying the quantitative and qualitative attributes of literature (14, 15). With the rapid expansion of scientific research, the importance of bibliometric analysis is increasingly prominent. Therefore, conducting bibliometric analysis is of profound significance for the study of disease evolution and cutting-edge trends. Currently, we have not found any bibliometric analysis on CBT in chronic pain research. This study aims to conduct a bibliometric analysis of research related to CBT in chronic pain over the past 20 years, specifically including the number of papers, major contributing countries, institutions, journals, and individuals. We aim to analyze the current research hotspots in the field, with the expectation of providing important reference evidence for researchers in the area of CBT for chronic pain.

## Materials and methods

### Data source and search strategy

**FIGURE 1:**
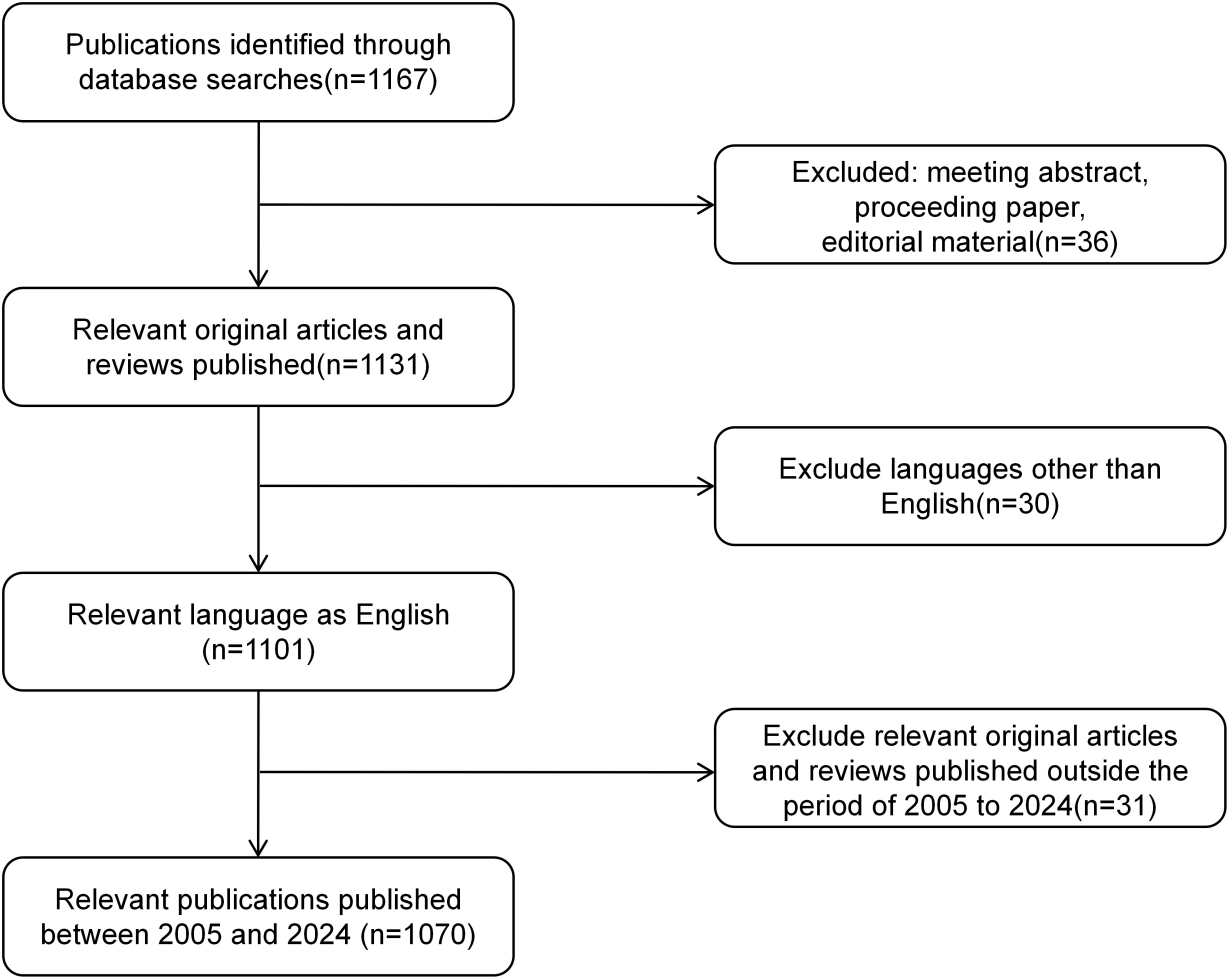
The inclusion and exclusion of publications on chronic pain with cognitive behavioral therapy.

This study retrieved literature related to chronic pain and cognitive behavioral therapy from the Web of Science Core Collection (WoSCC) database. To reduce the impact of database updates on data bias, literature retrieval and data extraction were completed on the same day. The search query was TS = “cognitive behavioral therapy” AND “chronic pain,” with the document types limited to articles or reviews, the language limited to English, and the time span from January 1, 2005, to December 31, 2024. After excluding duplicate articles, early online publications, incomplete articles, conference abstracts, and articles irrelevant to the topic, a total of 1,070 articles were included in this study. The specific process is shown in Figure 1. In addition, while WoSCC was used for visualization mapping, the PubMed database was independently searched to specifically retrieve and analyze clinical trial records. In PubMed, the search was limited to the publication type “Clinical Trial” using the query Abstract/Title = “cognitive behavioral therapy” AND “chronic pain”; other search parameters were the same as described above.

### Bibliometric analysis

This study is dedicated to comprehensively analyzing the academic publishing ecosystem, focusing on the dynamics of academic participation at the institutional and national levels. By utilizing the advanced analysis functions of the WoSCC database, we will delve into the multi-dimensional characteristics of academic output. During the literature classification process, the research team adopted a rigorous methodology, conducting a detailed assessment of each academic achievement in the dataset. Researchers accurately determined the academic type of the literature through systematic review of titles and abstracts, ensuring the accurate classification of “articles” and “reviews” based on in-depth analysis of the content and academic boundaries of each publication. The specific research path includes analyzing the academic participation patterns of countries and institutions, tracking annual research output trends, quantifying academic publication scale, organizing the distribution of core journals, and mapping knowledge in the research field. The “Analyze Results” function of the WoSCC database will serve as our key tool for extracting and processing academic information. We exported the data of 1,070 documents from the WoSCC database in the form of plain text files, named download_xx.txt. Excel 2019 and visualization analysis tools VOSviewer, CiteSpace, and the bibliometrix package were used to perform overall trend analysis, synonym merging, and cluster analysis of countries/regions, institutions, authors, keywords, and timeline charts to assess the research frontiers and hotspots of cognitive behavioral therapy in chronic pain. Data extraction and analysis were performed independently by two researchers. If the results were inconsistent, a third researcher participated in the discussion and reached a unified result.

Furthermore, this study deeply examines the dynamic evolution of academic publishing. By systematically investigating publication trends over time, and utilizing the impact factors and category quartile data from the 2024 Journal Citation Reports, the academic ecology of publications is analyzed from multiple perspectives using the R language bibliometric package. Microsoft Excel 2019, as the foundational platform for data management and processing, provided solid technical support for the research. We strategically employed VOSviewer and CiteSpace, two major visualization analysis tools, aiming to comprehensively present the network structure and knowledge map of academic research. These two software packages have their own characteristics in bibliometric analysis: VOSviewer focuses on revealing the citation relationships and co-occurrence patterns between publications, countries, institutions, and authors, while CiteSpace excels at capturing citation bursts and keyword clustering trends in the literature, accurately locating the key context and evolution path of academic research through visualization, providing an innovative perspective for a systematic understanding of the academic ecosystem (16–18).

Before formal analysis, standardized data preprocessing was performed on the 1,070 exported documents: we manually checked and merged synonymous items of author names, institutional affiliations and keywords, corrected spelling variations, and excluded invalid records with missing core information to ensure data consistency and result reliability. Core parameter settings of the visualization software were uniformly standardized for reproducible research: for CiteSpace 6.2.R3, the analysis time window was set to 2005–2024 with a 1-year time slicing interval, node selection followed the default Top 10 per slice algorithm, threshold parameters adopted the software’s recommended default values (LRF = 3.0, L/*N* = 10, LBY = 5, e = 1.0), pruning was set to None, and burst detection based on the Kleinberg algorithm was performed with a minimum burst duration of 2 years; for VOSviewer, the node inclusion threshold was set to a minimum occurrence frequency of 5, network normalization used the default Association strength method, clustering adopted the default modularity optimization algorithm with a resolution of 1.0, and three standard visualization modes (Network, Overlay, Density) were applied to present collaboration networks and topic distribution characteristics. Quantitative bibliometric indicator calculation was completed via the R-based bibliometrix package, including annual publication trend fitting, core journal Bradford distribution test, H-index statistics of countries/institutions/authors, and stratified analysis of journal influence combined with 2024 Journal Citation Reports data. Full-process quality control was implemented throughout the study: all data extraction and analysis were independently completed by two trained researchers with fully consistent parameter settings, and inconsistent results were resolved through in-depth discussion with a third senior researcher to ensure the rigor and repeatability of the research results.

## Results and discussion

### Annual publications and citation analysis

**FIGURE 2:**
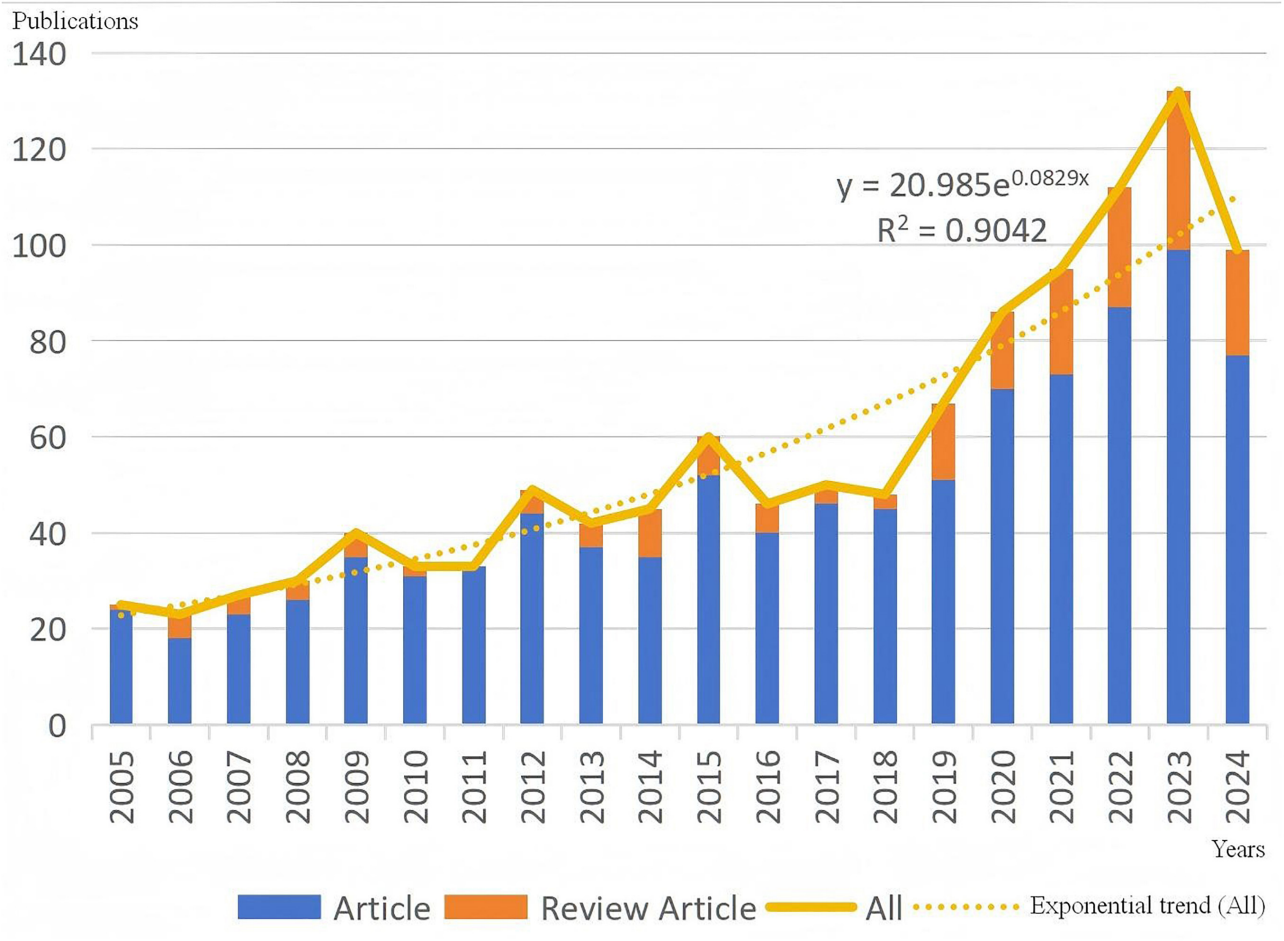
Annual number of publications on chronic pain with cognitive behavioral therapy.

This study included a total of 1,070 articles related to cognitive behavioral therapy in chronic pain published between 2005 and 2024. Among them, 743 (69.44%) were research articles and 327 (30.55%) were review articles. From 2005 to 2024, the cumulative number of articles published annually grew rapidly, presenting an exponential growth trend, with the specific formula being y = 20.985e^0.0829x^, *R*^2^ = 0.9042. The results are shown in Figure 2, where the horizontal axis shows the year and the vertical axis shows the number of articles published each year.

The total citations (TC) for these papers were 46,378, the average citations per item (ACI) was 43.26, and the H-index was 107. The trend of the number of publications and citation frequency over the past 20 years, indicating an overall upward trend in both areas, especially after 2021. Specifically, the average annual publications and citation frequency from 2005 to 2008 were 9 and 895.5, respectively, while the average annual publications and citation frequency from 2021 to 2024 were 96.25 and 1082.5, representing increases of 10.7 times and 1.2 times, respectively. These data indicate that in recent years, the research field of cognitive behavioral therapy in chronic pain not only maintains a high citation frequency but also has an increasing number of studies, continuously attracting widespread attention from many researchers, generating significant research interest, and achieving a large number of research results.

### Country/region analysis

**FIGURE 3:**
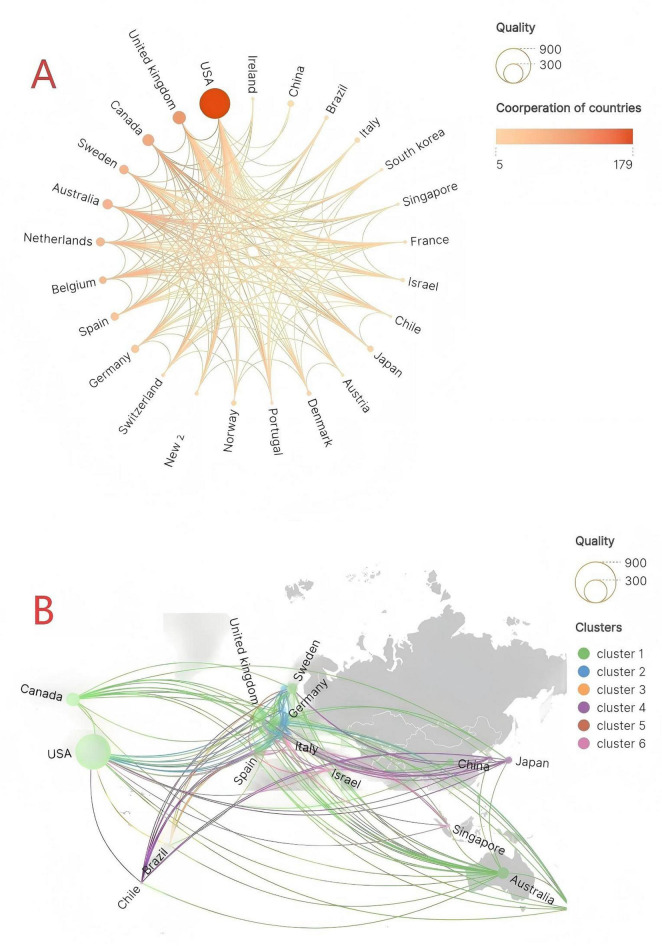
**(A)** Global cooperation network: geographical and intensity mapping of international collaboration. **(B)** Geographical distribution map of countries/regions cooperation.

A total of 67 countries/regions participated in the publication of these research papers. Among them, the United States (633 articles, 59.16%) was the most productive country, followed by the United Kingdom (109 articles, 10.19%) and Canada (93 articles, 8.69%). The combined publications of these three countries accounted for more than 78% of the total, making them leaders in the field. Table 1 shows the number of publications, publication share, average citations per paper, H-index, and centrality for the top 10 producing countries. It can be seen that the country/region with the highest average citations per paper is the United Kingdom, the country/region with the highest H-index is the United States, and the country/region with the highest centrality is the United Kingdom, indicating that the United States has made a greater contribution to the research of cognitive behavioral therapy in chronic pain. The network map in Figure 3A shows a complex international cooperation system, connecting nearly 30 countries from different continents through fine lines. The chart, centered on the United States, presents a gradient orange network, reflecting the breadth and depth of cooperation between countries. The legend indicates the cooperation strength (5–179) and quality indicators (300–900), and the density and color depth of the lines suggest the closeness of the connections between different countries. From a geographical distribution perspective, the figure includes multiple countries in North America, Europe, Asia, Oceania, and South America, such as the United States, United Kingdom, Germany, China, Japan, Australia, France, Canada, etc., reflecting the breadth and systematic nature of transnational collaboration in the context of globalization. This visualization method effectively presents the complexity and diversity of contemporary international cooperation networks, providing an intuitive image for understanding global interconnectedness. Figure 3B reveals the global academic network of cognitive behavioral therapy research in chronic pain through visualization analysis. Through cluster analysis, we observed 6 research clusters of different colors, with the green cluster dominated by the United States being particularly prominent, covering the widest range.

**TABLE 1:** Top 10 productive countries.

Rank	Country	Quantity	Proportion (%)	ACI	H-index	Centrality
1	United States	633	59.16%	44.88	85	0.31
2	United Kingdom	109	10.19%	58.98	43	0.44
3	Canada	93	8.69%	30.70	28	0.11
4	Australia	67	6.26%	45.40	28	0.05
5	Sweden	59	5.51%	40.70	24	0.11
6	Netherlands	53	4.95%	45.87	22	0.04
7	Spain	46	4.30%	33.87	19	0.11
8	Germany	43	4.02%	44.63	23	0.01
9	Belgium	37	3.46%	42.27	18	0.02
10	China	29	2.71%	46.31	14	0.06

### Institutional analysis

A total of 1,584 institutions participated in the publication of these 1,070 documents, among which the University Of Washington in the United States (106 articles, 9.91%) contributed the most documents, followed by the Seattle campus of the University Of Washington in the United States (105 articles, 9.81%) and the US Department of Veterans Affairs in the United States (100 articles, 9.35%). Detailed data in Table 2 shows that American institutions account for 9 of the top 10 producing institutions. The University Of Washington not only has high output, but also ranks first in terms of total citations (5,509) and H-index (38), indicating that its published papers are valued and widely cited by many researchers, and it is a leading core research institution. However, although the University Of Washington has the largest number of published academic papers and a high total number of citations and H-index, the University Of London in the United Kingdom has the highest average number of citations, as shown in detail in Table 2.

**TABLE 2:** Top 10 productive institutions.

Rank	Institution	Country	Quantity	SOTC	ACI	H-index
1	University of Washington	United States	106	5,509	51.97	38
2	University of Washington Seattle	United States	105	5,509	52.47	38
3	US Department of Veterans Affairs	United States	100	3,523	35.23	31
4	Harvard University	United States	96	5,146	53.60	33
5	Veterans Health Administration	United States	96	3,507	36.53	31
6	Harvard University Medical	United States	66	4,112	62.30	30
7	Harvard Medical School	United States	54	2,439	45.17	24
8	University Of California System	United States	53	2,322	43.81	20
9	Yale University	United States	43	1,912	44.47	20
10	University of London	United Kingdom	41	2n630	64.15	25

**FIGURE 4:**
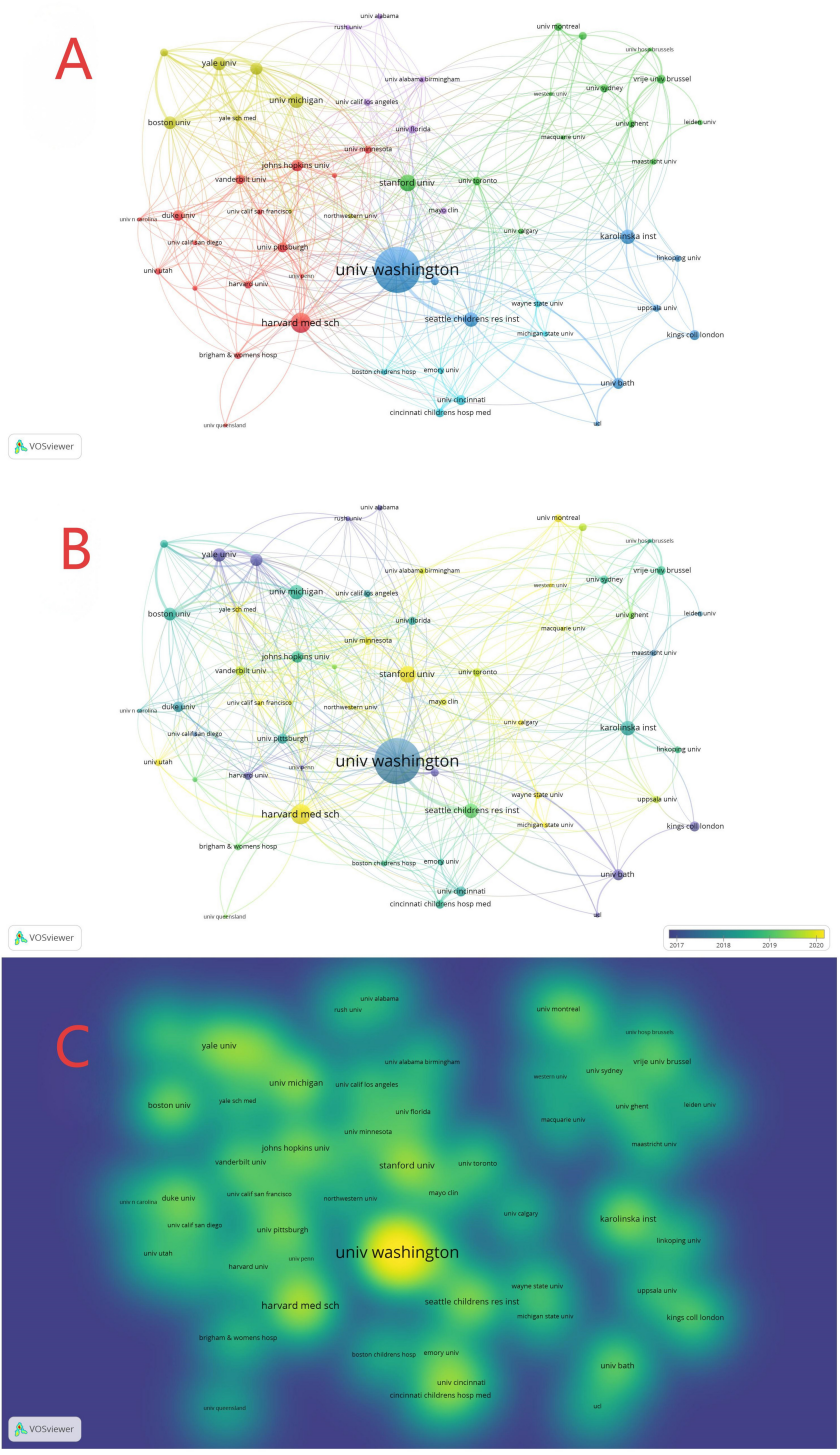
**(A)** Institutional collaboration network map. **(B)** Temporal evolution network map. **(C)** Collaboration density heatmap.

Based on VOSviewer visualization analysis, the complexity and dynamics of the global academic cooperation network are presented through institutional collaboration network diagrams, time evolution network diagrams, and thermal density maps. Figure 4A reveals the direct cooperative relationships between institutions, Figure 4B shows the evolution of cooperation over time, and Figure 4C highlights the dense areas and weak links in the research network. These visualization maps collectively reveal the central position of top American institutions represented by the University of Washington, Harvard University (see Supplementary material 1 for the full list), in global academic collaboration, reflecting not only the high concentration of academic resources, but also exposing structural imbalances in international academic cooperation. The world’s top academic productivity institutions exhibit significant geo-academic concentration, with American institutions occupying 9 positions on the list and only the University of London representing the United Kingdom in the top 10, reflecting the high regional concentration of academic innovation and research resources in this field. This singularity of academic geographical distribution not only highlights the dominance of the United States in this field, but also exposes the marginalization of emerging countries and regions in high-end academic production.

### Author analysis

A total of 4,820 authors participated in the publication of research papers, among which Palermo, Tonya M. (38 articles, 4.10%) from Seattle Children’s Hospital Ctr Child Hlth Behav and Dev in the United States is the most published author, followed by McCracken, Lance M. (38 articles, 4.10%) from Uppsala University Disciplinary Domain of Humanities and Social Sciences in the Sweden and Jensen, Mark P. (38 articles, 4.10%) from University of Washington UW Medicine in the United States. Table 3 shows detailed information for the top 10 authors ranked by number of publications. All of these authors are from the United States. Fisher, Emma has the highest average number of citations per paper (104.53) in the field, and McCracken, Lance M. has the highest H-index (22). Dr. Tonya Palermo is a professor of anesthesiology and pain medicine at the University of Washington, and also serves as an adjunct professor of pediatrics and psychiatry. Her research focuses on the behavioral, psychosocial, and family factors that influence the pain experience, the interrelationship between sleep and pain, and innovative psychotherapeutic methods for managing and preventing chronic pain (19). Dr. Lance McCracken is currently a professor of clinical psychology at Uppsala University and a visiting professor at the Department of Health Psychology at King’s College London. His research focuses on the development of treatments for chronic pain and psychological flexibility (20). Dr. Mark Jensen is a professor of rehabilitation medicine at the University of Washington. As a clinician/scientist, he has been dedicated to developing and researching the efficacy of psychosocial pain treatments for 30 years, focusing on the development and evaluation of pain, pain beliefs, and pain coping strategies, as well as the development and evaluation of psychological pain interventions. He served as editor-in-chief of The Journal of Pain for 12 years (21). Detailed results are shown in Table 3.

**TABLE 3:** Top 10 authors.

Rank	Author	Country	Institution	Quantity	ACI	H-index
1	Palermo, Tonya M.	United States	Seattle Children’s Hospital Ctr Child Hlth Behav & Dev	38	53.29	21
2	McCracken, Lance M.	Sweden	Uppsala University Disciplinary Domain of Humanities and Social Sciences	29	76.52	22
3	Jensen, Mark P.	United States	University of Washington UW Medicine	26	36.50	15
4	Edwards, Robert R.	United States	Brigham & Women’s Hospital Dept Anesthesiol Perioperat & Pain Management	17	99.41	9
5	Kerns, Robert D.	United States	VA Connecticut Healthcare System Pain Res Informat Multimorbid & Educ Ctr	17	50.41	13
6	Kashikar-Zuck, Susmita	United States	Cincinnati Children’s Hospital Medical Center Div Behav Med & Clin Psychol	17	33.12	12
7	Fisher, Emma	United Kingdom	Churchill Hosp, Pain Res Unit, Palliat & Support Care Grp, Cochrane Pain	17	104.53	15
8	Law, Emily F.	United States	University of Kansas University of Kansas Medical Center	16	29.81	10
9	Keefe, Francis	United States	Duke University Duke Medicine	14	62.43	11
10	Nijs, Jo	Belgium	Vrije Universiteit Brussel, VUB, Free University of Brussels	13	61.77	9

**FIGURE 5:**
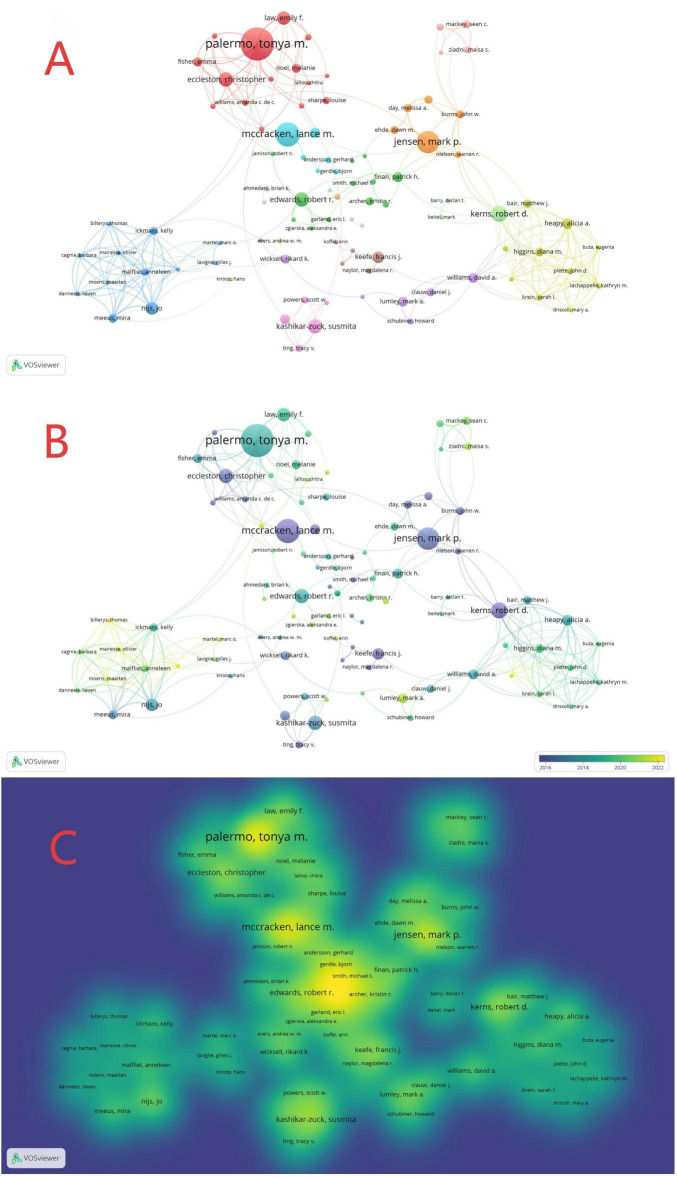
**(A)** Author’s collaboration network map. **(B)** Temporal evolution network map. **(C)** Collaboration density heatmap.

Figure 5A, the cluster view, uses color gradients to show the collaboration between highly cited authors with at least 50 citations. The areas centered on “Palermo, Tonya M.” and “Law, Emily F.” show a high-density yellow color, indicating that these authors and the scholars around them have formed a core research community or collaboration network in the field, with a high degree of consistency in research topics or methods. In contrast, the blue areas in the figure represent relatively isolated researchers. Figure 5B, the label view, uses nodes to represent authors, lines between nodes to represent collaboration or citation relationships, and node colors to encode the time information of authors’ activity in the field. The color gradually changes from dark to light, with dark colors representing earlier research activities and light colors representing the latest research results. Therefore, the clusters in the figure not only reflect the collaboration between authors, but also reveal the evolution of research topics over time. Figure 5C shows the research activity profile in the field, and several prominent high-density areas can be seen, especially around “Palermo, Tonya M.”, “McCracken, Lance M.”, “Jensen, Mark P.”, “Kerns, Robert D.”, “Edwards, Robert R.”, and “Kashikar-Zuck, Susmita,” indicating that these authors and their related networks are at the core of the research landscape in this field.

### Journal analysis

The relevant literature included in this study was published in 356 journals. Among them, *Journal of Pain* published the most papers (75 articles, 7.01%), followed by *Pain* (60 articles, 5.61%) and *Pain Medicine* (59 articles, 5.51%). Detailed information on the top 10 journals in terms of publication output is shown in Table 4. From the data in the table, it can be seen that the average number of citations for these journals is 41.73, and most journals belong to the Q1 or Q2 category, indicating that most of the research results in this field are published in journals with moderate impact, which to some extent reflects the significant potential for improving the quality of research in this field. In addition, it is worth noting that most of these high-output journals originate from the United States or Europe, which indicates that journals in these regions have made important contributions to knowledge dissemination. The *Journal of Pain* (86.67), which has the highest average number of citations, is one of the top and most influential journals in the field of pain research, with a very high reputation and academic status. *Journal of Pain* is the official journal of the U.S. Association for the Study of Pain (USASP). *Journal of Pain* has a wide readership and authority in the field of pain research. USASP is one of the largest academic organizations in the field of global pain research, representing a community of professionals, scientists, and policymakers dedicated to advancing the development of pain research, and serves as an important professional association. As a key force in the field of pain research, USASP regularly holds annual meetings to provide participants with a platform to exchange cutting-edge research results in pain science and management. *Journal of Pain* publishes original research related to all aspects of pain, including the mechanisms of pain, neurobiological basis, psychological and social factors, clinical research on acute and chronic pain, various pain treatment methods, pain assessment tools and methods, and pain research in special populations (such as children or the elderly).

**TABLE 4:** Top 10 journals.

Rank	Journal	Quantity	ACI	IF (2023)	JCR
1	Journal of Pain	75	67.12	4.00	Q1
2	Pain	60	86.87	5.90	Q1
3	Pain Medicine	59	27.98	2.90	Q1
4	Clinical Journal of Pain	56	41.80	2.60	Q2
5	Current Pain and Headache Reports	26	18.81	3.20	Q2
6	Trials	21	5.48	2.00	Q3
7	European Journal of Pain	20	59.35	3.50	Q1
8	Contemporary Clinical Trials	19	7.26	2.00	Q3
9	Journal of Pain research	18	28.61	2.50	Q2
10	Journal of medical Internet Research	17	34.06	5.80	Q1

### Keyword analysis

**FIGURE 6:**
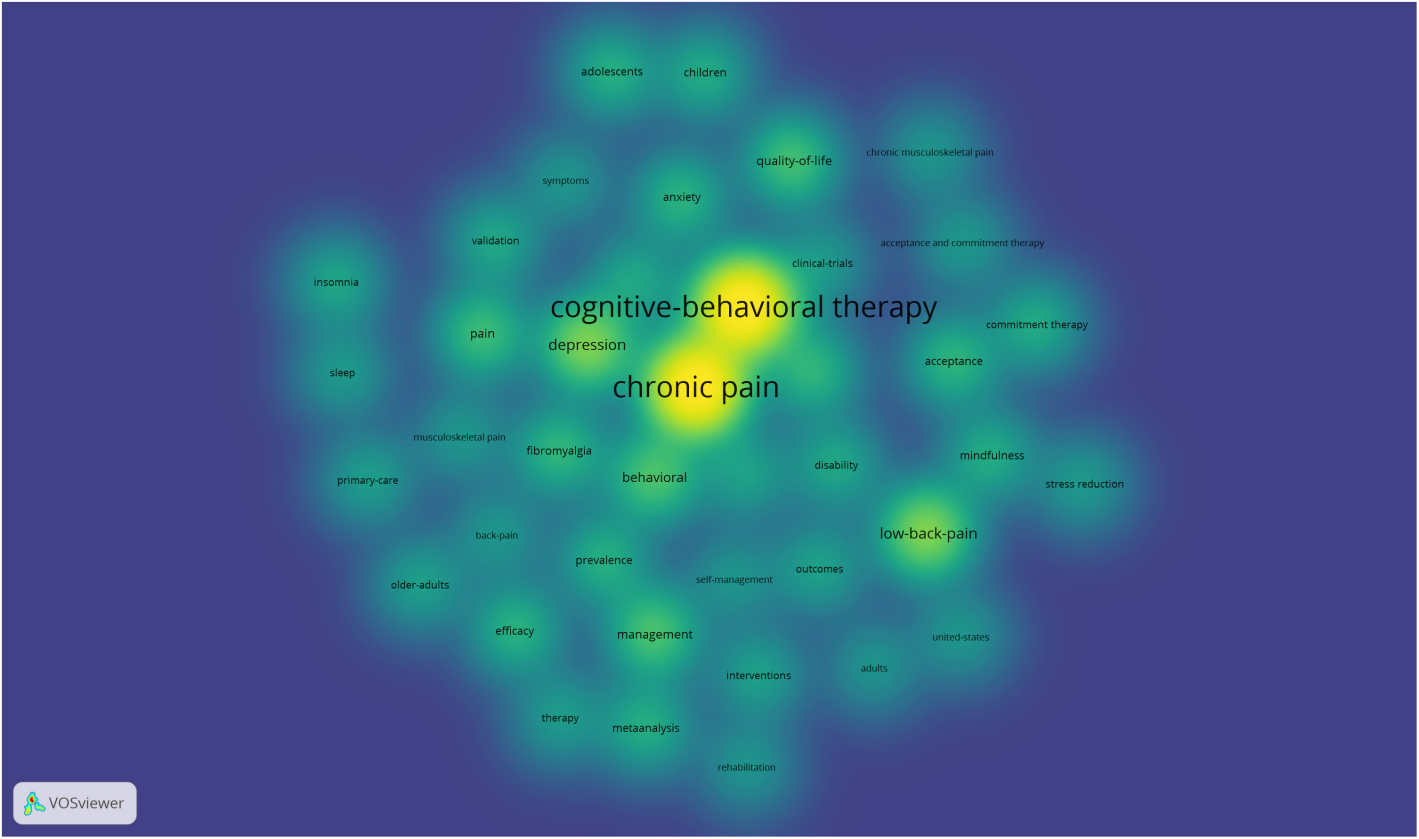
Keywords collaboration density heatmap.

Keywords are highly condensed summaries of the research topics in a paper. By analyzing high-frequency keywords, one can gain an in-depth understanding of the main research content and hot research topics in a specific field. Table 5 lists the top 20 high-frequency keywords, and Figure 6 shows the clustering relationship between keywords that appear at least 50 times. These high-frequency keywords provide an overall overview of the main research content in this field. Each circle in the figure represents a keyword, and the larger the node, the higher the frequency or importance of the keyword in the research field. Chronic Pain and Cognitive Behavioral Therapy are the center of the figure, indicating that cognitive behavioral therapy plays an important role in chronic pain management. Low-back Pain is another important topic related to chronic pain and cognitive behavioral therapy, and is related to chronic pain and cognitive behavioral therapy. The figure also includes other keywords such as Depression, Sleep, Anxiety, and Quality of Life, which are closely related to chronic pain.

**TABLE 5:** Top 20 keywords.

Rank	Keywords	Count	Centrality
1	Cognitive behavioral therapy	726	0.05
2	Chronic pain	662	0.05
3	Low back pain	238	0.09
4	Depression	165	0.04
5	Quality of life	151	0.03
6	Management	145	0.06
7	Randomized controlled trial	145	0.02
8	Prevalence	95	0.07
9	Efficacy	93	0.03
10	Metaanalysis	93	0.04
11	Acceptance	91	0.05
12	Commitment therapy	87	0.02
13	Primary care	86	0.08
14	Older adults	79	0.03
15	Validation	79	0.02
16	Adolescents	74	0.02
17	Disability	71	0.01
18	Children	70	0.04
19	Outcomes	67	0.01
20	Interventions	66	0.05

**FIGURE 7:**
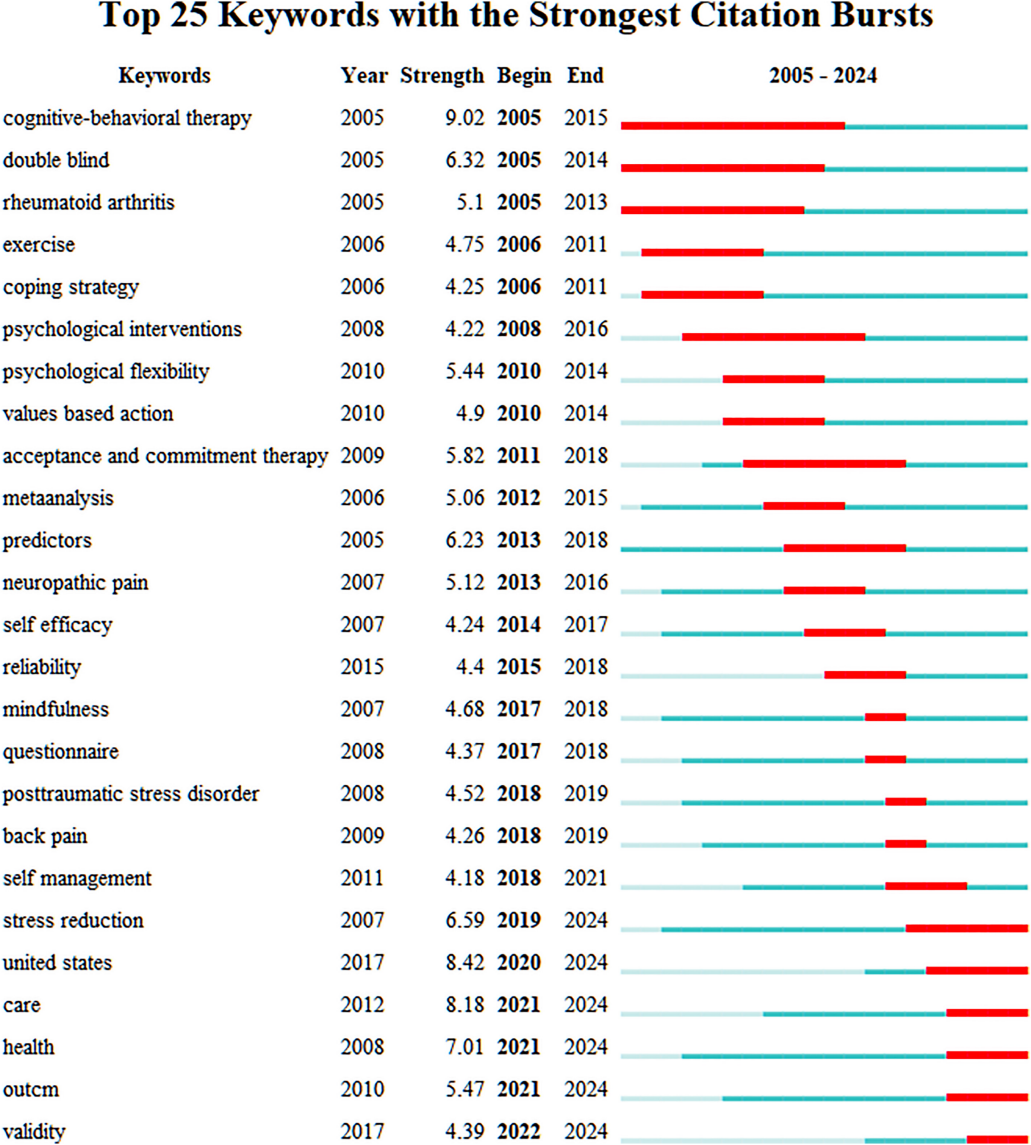
Top 25 keywords with the strongest keyword bursts.

Keyword burst detection can indicate current hot research topics and trends in a field, as shown in Figure 7. Red bars indicate the start, end, and duration of citation bursts. “Cognitive-behavioral therapy,” “double blind,” and “rheumatoid arthritis” are the earliest keywords to appear, with an average burst intensity of 6.81 and a duration of nearly 10 years, indicating that it was a topic of great interest in the early stages. “Cognitive-behavioral therapy” also has the longest duration, appearing in 2005 and lasting for 10 years, and is a topic of continuous attention by researchers. Excluding the subject terms and free words of CBT, “stress reduction,” “double blind,” and “predictors” are keywords with higher burst intensity, suggesting that in addition to CBT, more attention is paid to intervention research using stress relief strategies, strict requirements for research design methodology (double-blind), and exploration of potential predictors in problems such as chronic pain are important directions for future research. “Stress reduction,” “united states,” “care,” “health,” “outcomes,” and “validity” indicate that current research trends continue to focus on stress relief methods, research in the United States is still active in this field, and at the same time, high attention is paid to caring medical care, overall health, the reliability of research results, and the validity of the research itself. These elements together constitute the focus of research in this field.

**FIGURE 8:**
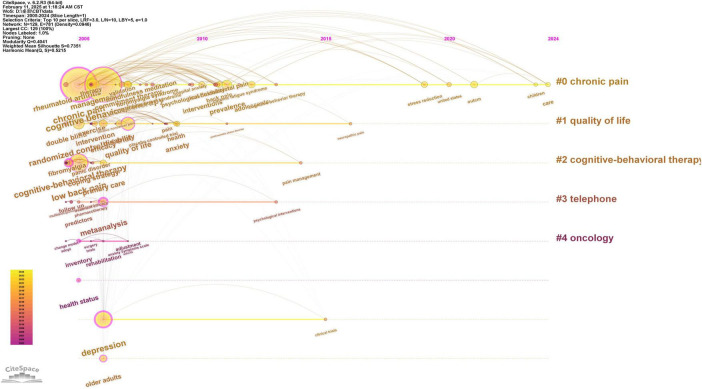
CiteSpace visualization timeline view of keywords clustering analysis.

CiteSpace clusters keywords and presents them in a timeline view; Figure 8 shows the clustering results of the top five clusters. Horizontal lines of different colors represent clusters formed by keywords, nodes on the horizontal lines represent keywords, and the position of the nodes represents the year in which the literature containing the keywords first appeared. “Chronic pain” is the largest keyword cluster, followed by “quality of life.” Excluding the subject terms and free words of CBT, the two clusters “telephone” and “oncology” indicate that, in addition to chronic pain and quality of life, the use of telephone follow-up methods and attention to pain management in cancer patients are directions worthy of attention in this field.

### Analysis of highly cited references

Highly cited references not only indicate high-quality research results, but also reflect the basic knowledge of a specific research field. Table 6 presents the top 20 cited references. Beyond equating citation counts with importance, analyzing why these works became highly cited reveals how they introduced conceptual innovations, resolved controversies, or consolidated existing knowledge.

**TABLE 6:** Top 20 cited references.

Rank	Title	Journal	Author	Year	Citation
1	Fibromyalgia a clinical review	JAMA-Journal of the American Medical Association	Clauw, DJ	2014	1,100
2	The Association of sleep and pain: an update and a path forward	JOurnal of Pain	Finan, PH	2013	982
3	Pain catastrophizing: a critical review	Expert Review of Neurotherapeutics	Quartana, PJ	2009	977
4	Mindfulness interventions	Annual Review of Psychology	Creswell, JD	2017	882
5	Fifteen years of explaining pain: the past, present, and future	Journal of Pain	Moseley, GL	2015	501
6	Acceptance-based interventions for the treatment of chronic pain: A systematic review and meta-analysis	Pain	Veehof, MM	2011	482
7	Anxiety disorders and comorbid medical illness	General Hospital Psychiatry	Roy-Byrne, PP	2008	459
8	The link between depression and chronic pain: neural mechanisms in the brain	Neural Plasticity	Sheng, JY	2017	433
9	Role of the prefrontal cortex in pain processing	Molecular Neurobiology	Ong, WY	2019	421
10	Fibromyalgia syndrome: Review of clinical presentation, pathogenesis, outcome measures, and treatment	Journal of Rheumatology	Mease, P	2005	391
11	Mediators, moderators, and predictors of therapeutic change in cognitive-behavioral therapy for chronic pain	Pain	Turner, JA	2007	387
12	Acceptance and commitment therapy (ACT) for chronic pain: a systematic review and meta-analyses	Clinical Journal of Pain	Hughes, LS	2017	384
13	Chronic pain and mental health disorders: shared neural mechanisms, epidemiology, and treatment	Mayo Clinic Proceedings	Hooten, WM	2016	354
14	The psychological flexibility model: a basis for integration and progress in psychological approaches to chronic pain management	Journal of Pain	McCracken, LM	2014	353
15	A randomized, controlled trial of acceptance and commitment therapy and cognitive-behavioral therapy for chronic pain	Pain	Wetherell, JL	2011	347
16	Cognitive behavioral therapy for insomnia comorbid with psychiatric and medical conditions a meta-analysis	Jama Internal Medicine	Wu, JQ	2015	335
17	Evaluating psychosocial contributions to chronic pain outcomes	Progress in Neuro-Psychopharmacology & Biological Psychiatry	Meints, SM	2018	302
18	Sleep deficiency and chronic pain: potential underlying mechanisms and clinical implications	Neuropsychopharmacology	Haack, M	2020	276
19	Pain and depression: a systematic review	Harvard Review of Psychiatry	IsHak, WW	2018	266
20	Psychological processing in chronic pain: A neural systems approach	Neuroscience and Biobehavioral Reviews	Simons, LE	2014	260

The most cited paper, a clinical review of fibromyalgia by Clauw (22), provided an authoritative synthesis that firmly established central sensitization as a core mechanism and CBT as a cornerstone of management (22, 23). Finan’s update on the sleep–pain relationship challenged unidirectional causality and delineated a biopsychosocial research agenda (24). Quartana’s critical review positioned pain catastrophizing as a central psychological mechanism, legitimizing CBT as the primary targeted intervention (25). Creswell’s review of mindfulness integrated cognitive-behavioral and third-wave mechanisms, catalyzing the shift toward process-oriented chronic pain care (26). Moseley’s Explain Pain framework reframed pain as a protective response, inspiring pain neuroscience education (27). A subsequent meta-analysis by Veehof et al. provided the first pooled effect-size estimates for acceptance-based interventions against CBT, demonstrating moderate yet comparable efficacy (28). A further meta-analysis by Hughes et al. empirically validated ACT’s unique therapeutic targets—pain acceptance and psychological flexibility—without showing superiority in pain intensity reduction (29). Wu et al.’s meta-analysis of CBT for comorbid insomnia established robust transdiagnostic efficacy on both sleep and co-occurring symptoms, supporting its integration into chronic pain management (30).

Overall, the highly cited references collectively define the intellectual structure of chronic pain research. Foundational reviews consolidated the biopsychosocial framework (23, 31–33), while cumulative meta-analytic benchmarks progressively traced the paradigm shift from traditional CBT toward ACT and mindfulness (29, 34, 35). Core transdiagnostic constructs—pain catastrophizing and psychological flexibility—were empirically established as key mediators (20, 36, 37). Bidirectional sleep–pain models fostered the development of integrated treatment approaches (30, 38), and neurobiological reviews bridged neuroscience with psychological models (39). Together, systematic reviews and meta-analyses supplied quantitative effect-size benchmarks that now anchor clinical guidelines.

### Citation burst analysis

**FIGURE 9:**
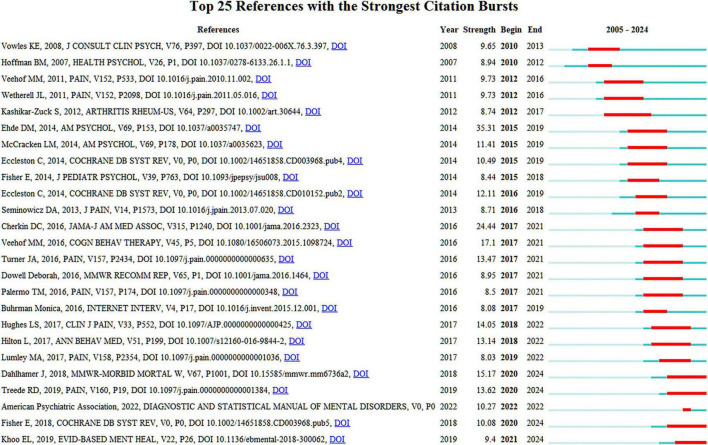
Top 25 references with the strongest citation bursts.

Citation burst refers to the phenomenon where certain papers are frequently cited within a specific period. By analyzing burst citations, we can understand the hot research topics of that period. In this study, we detected 25 references with high burst intensity, as shown in Figure 9. In the figure, “Intensity” represents the intensity of the citation burst, with higher values indicating stronger bursts. “Begin” and “End” indicate the start and end times of the burst, respectively, with the blue line representing the time interval and the red line representing the duration of the burst. Figure 9 shows the top 25 references with the highest citation burst intensity, reflecting their influence in the field. Notably, Ehde (6) has the highest citation intensity (35.31), reflecting its significant impact and continued relevance from 2015 to 2019. This article discusses CBT’s effectiveness, including innovative modalities like online and group therapy, and calls for research on cultural adaptation and combined treatments (10). Cherkin (40) also showed a significant citation burst, peaking from 2017 to 2021. This RCT demonstrated that both MBSR and CBT led to greater improvements in functional limitations and pain bothersomeness compared to usual care, supporting psychosocial interventions for chronic low back pain (41). These citation bursts likely reflect a paradigmatic shift in pain science from a predominantly biomedical model toward biopsychosocial and integrative frameworks. This transition was spurred by refined pain phenotyping—including distinctions among nociceptive, neuropathic, and nociplastic pain—and increased recognition of central mechanisms and maladaptive neuroplasticity even in the absence of tissue damage. Such conceptual advances have promoted broader acceptance and expansion of CBT and third-wave therapies in chronic pain management.

Furthermore, it can be seen from the figure that the burst duration of 4 references has continued from 2020 to the present. These papers, to some extent, reflect recent research hotspots and future development trends. Therefore, subsequent analysis will focus on these selected literatures. A CDC study revealed that approximately 20.4% of U.S. adults have chronic pain, with higher prevalence among socioeconomically disadvantaged groups, underscoring the need for targeted interventions (5). A randomized controlled trial comparing blended emotion-focused therapy to iCBT found that the transdiagnostic approach improved pain interference and catastrophizing, with higher adherence (42). A Cochrane review of 47 trials concluded that psychological therapies, primarily CBT, may reduce pain frequency in pediatric headache, but effects were not sustained (43). A network meta-analysis of 21 RCTs found small effect advantages for both MBSR and CBT over controls on physical function and pain intensity, with no significant difference between them, highlighting the need for rigorous comparison studies (44). These sustained bursts mirror the field’s deepening engagement with the biopsychosocial model, as researchers target high-burden populations, compare transdiagnostic and mindfulness-based interventions, and evaluate long-term outcomes and adherence—reflecting a shift toward personalized, mechanism-informed chronic pain care.

### Strengths and limitations

This study uses a bibliometric approach to comprehensively summarize and analyze relevant literature on cognitive behavioral therapy in chronic pain and presents the research results in the form of visualized knowledge maps. Compared with traditional literature reviews or meta-analyses, this study includes more comprehensive literature, more detailed data processing and analysis, and is not affected by subjective selection and judgment, thus presenting more objective and true research results. However, even so, this study still has certain limitations. First, the literature data included in this study were retrieved from the Web of Science Core Collection (WoSCC) and PubMed databases. Although the integration of multiple databases enhances the comprehensiveness of the data, the inclusion criteria were still restricted to English-language publications and specific document types (original articles and reviews). This may lead to the omission of some literature that meets the inclusion criteria but is published in other languages or indexed in different databases. Notably, the language restriction may introduce a structural bias toward English-speaking countries (such as the United States, United Kingdom, and Canada), potentially resulting in a geographic distribution that reflects the English-language research ecosystem rather than the full global scientific landscape. However, such operations ensure the high quality, standardization, and professional relevance of the data, providing a robust foundation for bibliometric visualization. Second, because some recently published high-quality research results have low citation rates, their academic influence may be underestimated. However, the results of this study analysis still provide important reference value and guidance for scholars in the field. This bibliometric analysis reveals the research hotspots, development trends, and major contributors in the field of cognitive behavioral therapy for chronic pain, providing important reference directions for medical and psychological practitioners.

### Clinical advances

Integrating the themes from the preceding citation burst and highly cited reference analyses, current clinical research on CBT for chronic pain can be characterized by several systematic advances that directly extend earlier conceptual and empirical shifts.

(1) Digitization and accessibility, foreshadowed by the proliferation of internet-based trials in highly cited meta-analyses and the population-level data on chronic pain burden, has become a core direction. Research has widely shifted from in-person therapy to iCBT, mHealth, and telehealth, with non-inferiority demonstrated for pain interference, function, and psychological outcomes (45–47). This addresses access barriers and enables AI integration and personalized feedback (48).

(2) Evolution toward third-wave therapies, anticipated by citation bursts around MBSR/CBT trials and foundational reviews of mindfulness and ACT, is expanding the therapeutic armamentarium. ACT shows comparable or superior efficacy to CBT, particularly in pain acceptance (49), while emerging EAET has demonstrated greater pain reduction than CBT in older adults (50, 51), mindfulness-CBT integration continues to receive attention (52).

(3) Integrated comorbidity management, aligned with the bidirectional sleep–pain models and CBT-I meta-analyses identified as highly cited, has become the norm. CBT-I robustly improves sleep and indirectly reduces pain and fatigue (53–55). Amid the opioid crisis, CBT assists opioid tapering and manages substance use risk (56–58), and for pain with depression/anxiety, CBT yields dual benefits (40, 59, 60).

(4) Precision and personalization, building on earlier mediator/moderator studies highlighted among highly cited references, aim to predict “for whom it works.” Baseline catastrophizing, fear-avoidance (13), neuroimaging biomarkers (61, 62), and quantitative sensory testing (63) are used to forecast response. Literacy-adapted, culturally tailored (64–66), and condition-specific protocols have been validated (67, 68).

(5) Mechanistic investigation, consistent with neurobiological reviews among highly cited references, confirms that CBT modulates prefrontal-limbic networks (69), Process research identifies adherence, skill practice, early response, and therapeutic alliance as key mediators (70, 71).

(6) Lifespan and setting expansion reflects the population-level vision implied by cited foundational reviews and surveillance data. Applications now span children and adolescents (72–74) to older adults, include perioperative prevention (75, 76), and extend into primary care (77) and communities via trained nurses (78) or peers (79, 80), addressing health equity and implementation.

In summary, these clinical advances are directly translating the intellectual structure revealed by bibliometric analysis—from biopsychosocial consolidation to third-wave integration—into digitalized, personalized, and mechanism-informed chronic pain care.

## Conclusion

In the past 20 years, research related to cognitive behavioral therapy in chronic pain has experienced rapid development, especially since 2021, when a large number of academic achievements in the field have emerged and been widely disseminated and cited. The United States has become the most productive country in the field, while the University of Washington has contributed the most research results, and the research results of the University of London are more popular among scholars. The *Journal of Pain* published the most papers, while Pain became the most influential journal. Keywords such as Depression, Sleep, Anxiety, and Quality of Life reflect the current hotspots and future frontier trends in the field. The future trend of CBT in the treatment of chronic pain is to apply it in a more personalized and comprehensive manner, and to combine the latest research results in psychology, neurobiology, etc. to improve the therapeutic effect.

## Data Availability

The raw data supporting the conclusions of this article will be made available by the authors, without undue reservation.
