# Supplementary material for: Emerging trends and hotspots in cognitive behavioral therapy for chronic pain: a bibliometric analysis
Source: Front Med (Lausanne). 2026 Jun 17;13:1788247. doi: 10.3389/fmed.2026.1788247 (PMC13318720; doi:10.3389/fmed.2026.1788247)
Supplement: Supplementary file 1 [file Table_1.docx]

| Affiliations | Count |
| --- | --- |
| UNIVERSITY OF WASHINGTON | 143 |
| UNIVERSITY OF WASHINGTON SEATTLE | 141 |
| US DEPARTMENT OF VETERANS AFFAIRS | 141 |
| VETERANS HEALTH ADMINISTRATION VHA | 136 |
| HARVARD UNIVERSITY | 134 |
| HARVARD UNIVERSITY MEDICAL AFFILIATES | 126 |
| HARVARD MEDICAL SCHOOL | 73 |
| UNIVERSITY OF CALIFORNIA SYSTEM | 73 |
| STANFORD UNIVERSITY | 58 |
| YALE UNIVERSITY | 57 |
| JOHNS HOPKINS UNIVERSITY | 56 |
| SEATTLE CHILDREN S HOSPITAL | 51 |
| STATE UNIVERSITY SYSTEM OF FLORIDA | 47 |
| UNIVERSITY OF MICHIGAN SYSTEM | 47 |
| UNIVERSITY OF MICHIGAN | 46 |
| BRIGHAM WOMEN S HOSPITAL | 44 |
| UNIVERSITY SYSTEM OF OHIO | 43 |
| VA CONNECTICUT HEALTHCARE SYSTEM | 42 |
| BOSTON UNIVERSITY | 39 |
| MASSACHUSETTS GENERAL HOSPITAL | 36 |
| PENNSYLVANIA COMMONWEALTH SYSTEM OF HIGHER EDUCATION PCSHE | 36 |
| UNIVERSITY OF ALABAMA SYSTEM | 35 |
| UNIVERSITY OF CALIFORNIA LOS ANGELES | 33 |
| UTAH SYSTEM OF HIGHER EDUCATION | 33 |
| DUKE UNIVERSITY | 31 |
| STATE UNIVERSITY OF NEW YORK SUNY SYSTEM | 31 |
| CINCINNATI CHILDREN S HOSPITAL MEDICAL CENTER | 29 |
| UNIVERSITY OF CINCINNATI | 29 |
| UNIVERSITY OF FLORIDA | 28 |
| VANDERBILT UNIVERSITY | 28 |
| UNIVERSITY OF PITTSBURGH | 27 |
| UNIVERSITY OF TEXAS SYSTEM | 27 |
| VA BOSTON HEALTHCARE SYSTEM | 26 |
| UNIVERSITY OF UTAH | 25 |
| OREGON HEALTH SCIENCE UNIVERSITY | 24 |
| BOSTON CHILDREN S HOSPITAL | 23 |
| UNIVERSITY AT BUFFALO SUNY | 23 |
| UNIVERSITY OF MINNESOTA SYSTEM | 23 |
| UNIVERSITY OF MINNESOTA TWIN CITIES | 23 |
| MAYO CLINIC | 21 |
| DAVID GEFFEN SCHOOL OF MEDICINE AT UCLA | 20 |
| UNIVERSITY OF CALIFORNIA LOS ANGELES MEDICAL CENTER | 20 |
| UNIVERSITY OF CALIFORNIA SAN DIEGO | 20 |
| UNIVERSITY OF NORTH CAROLINA | 20 |
| INDIANA UNIVERSITY SYSTEM | 19 |
| UNIVERSITY OF ALABAMA BIRMINGHAM | 19 |
| UNIVERSITY OF PENNSYLVANIA | 19 |
| WAYNE STATE UNIVERSITY | 19 |
| MICHIGAN STATE UNIVERSITY | 18 |
| BROWN UNIVERSITY | 17 |
| EMORY UNIVERSITY | 17 |
| RUSH UNIVERSITY | 17 |
| UNIVERSITY OF ALABAMA TUSCALOOSA | 17 |
| UNIVERSITY OF CALIFORNIA SAN FRANCISCO | 17 |
| UNIVERSITY OF TORONTO | 17 |
| JOHNS HOPKINS MEDICINE | 16 |
| KAISER PERMANENTE | 16 |
| NORTHWESTERN UNIVERSITY | 16 |
| UNIVERSITY OF NORTH CAROLINA CHAPEL HILL | 16 |
| BETH ISRAEL DEACONESS MEDICAL CENTER | 15 |
| FEINBERG SCHOOL OF MEDICINE | 15 |
| MICHIGAN STATE UNIVERSITY COLLEGE OF HUMAN MEDICINE | 15 |
| KAROLINSKA INSTITUTET | 14 |
| UNIVERSITY OF MASSACHUSETTS SYSTEM | 14 |
| UNIVERSITY SYSTEM OF MARYLAND | 13 |
| BAYLOR COLLEGE OF MEDICINE | 12 |
| CHILDREN S HEALTHCARE OF ATLANTA CHOA | 12 |
| CORNELL UNIVERSITY | 12 |
| INDIANA UNIVERSITY BLOOMINGTON | 12 |
| MINNEAPOLIS VA HEALTH CARE SYSTEM | 12 |
| UNIVERSITY OF CONNECTICUT | 12 |
| UNIVERSITY OF NEW MEXICO | 12 |
| UNIVERSITY OF TEXAS AT SAN ANTONIO | 12 |
| WESTERN UNIVERSITY UNIVERSITY OF WESTERN ONTARIO | 12 |
| ICAHN SCHOOL OF MEDICINE AT MOUNT SINAI | 11 |
| UNIVERSITY OF BATH | 11 |
| UNIVERSITY OF CALGARY | 11 |
| UNIVERSITY OF COLORADO SYSTEM | 11 |
| UNIVERSITY OF MARYLAND BALTIMORE | 11 |
| UNIVERSITY OF OXFORD | 11 |
| UNIVERSITY OF WISCONSIN SYSTEM | 11 |
| VA SAN DIEGO HEALTHCARE SYSTEM | 11 |
| VIRGINIA COMMONWEALTH UNIVERSITY | 11 |
| WEILL CORNELL MEDICINE | 11 |
| ARIZONA STATE UNIVERSITY | 10 |
| LOUISIANA STATE UNIVERSITY SYSTEM | 10 |
| RUTGERS UNIVERSITY NEW BRUNSWICK | 10 |
| RUTGERS UNIVERSITY SYSTEM | 10 |
| UNIVERSITY OF MASSACHUSETTS WORCESTER | 10 |
| UNIVERSITY OF WISCONSIN MADISON | 10 |
| WASHINGTON UNIVERSITY WUSTL | 10 |
| CALIFORNIA STATE UNIVERSITY SYSTEM | 9 |
| GERIATRIC RESEARCH EDUCATION CLINICAL CENTER | 9 |
| HOSPITAL FOR SICK CHILDREN SICKKIDS | 9 |
| NEW YORK UNIVERSITY | 9 |
| OHIO STATE UNIVERSITY | 9 |
| PENNSYLVANIA STATE UNIVERSITY | 9 |
| UNIVERSITY OF ARIZONA | 9 |
| UNIVERSITY OF COLORADO ANSCHUTZ MEDICAL CAMPUS | 9 |
| UNIVERSITY OF ILLINOIS SYSTEM | 9 |
| UNIVERSITY OF LONDON | 9 |
| UNIVERSITY OF SOUTH FLORIDA | 9 |
| UNIVERSITY OF VERMONT | 9 |
| UNIVERSITY WESTERN ONTARIO HOSPITAL | 9 |
| UTAH STATE UNIVERSITY | 9 |
| VA ANN ARBOR CTR CLIN MANAGEMENT RES | 9 |
| BAYLOR COLLEGE MEDICAL HOSPITAL | 8 |
| MCGILL UNIVERSITY | 8 |
| MEDICAL UNIVERSITY OF SOUTH CAROLINA | 8 |
| NATIONAL INSTITUTES OF HEALTH NIH USA | 8 |
| REGENSTRIEF INSTITUTE INC | 8 |
| RICHARD L ROUDEBUSH VA MEDICAL CENTER | 8 |
| UNIVERSITY OF ILLINOIS CHICAGO | 8 |
| UNIVERSITY OF ILLINOIS CHICAGO HOSPITAL | 8 |
| UNIVERSITY OF IOWA | 8 |
| UNIVERSITY SYSTEM OF GEORGIA | 8 |
| UPPSALA UNIVERSITY | 8 |
| VA GREATER LOS ANGELES HEALTHCARE SYSTEM | 8 |
| VA PALO ALTO HEALTH CARE SYSTEM | 8 |
| WAKE FOREST UNIVERSITY | 8 |
| ANN ROBERT H LURIE CHILDREN S HOSPITAL OF CHICAGO | 7 |
| ARIZONA STATE UNIVERSITY TEMPE | 7 |
| CLEVELAND CLINIC FOUNDATION | 7 |
| CONNECTICUT CHILDREN S MEDICAL CENTER | 7 |
| DANA FARBER CANCER INSTITUTE | 7 |
| INDIANA UNIVERSITY INDIANAPOLIS | 7 |
| JAMES A HALEY VETERANS HOSPITAL | 7 |
| LIFESPAN HEALTH RHODE ISLAND | 7 |
| RUTGERS UNIVERSITY BIOMEDICAL HEALTH SCIENCES | 7 |
| THOMAS JEFFERSON UNIVERSITY | 7 |
| UNIVERSITY COLLEGE LONDON | 7 |
| UNIVERSITY OF ROCHESTER | 7 |
| UNIVERSITY OF VIRGINIA | 7 |
| COLUMBIA UNIVERSITY | 6 |
| CREIGHTON UNIVERSITY | 6 |
| DREXEL UNIVERSITY | 6 |
| GEORGETOWN UNIVERSITY | 6 |
| GEORGIA STATE UNIVERSITY | 6 |
| GRP HLTH RES INST | 6 |
| HARBORVIEW MEDICAL CENTER | 6 |
| HENRY FORD HEALTH SYSTEM | 6 |
| MAASTRICHT UNIVERSITY | 6 |
| NYU LANGONE MEDICAL CENTER | 6 |
| UNIFORMED SERVICES UNIVERSITY OF THE HEALTH SCIENCES USA | 6 |
| UNIVERSITE DE MONTREAL | 6 |
| UNIVERSITY OF KANSAS | 6 |
| UNIVERSITY OF LOUISVILLE | 6 |
| UNIVERSITY OF OSLO | 6 |
| UNIVERSITY OF QUEENSLAND | 6 |
| UNIVERSITY OF SOUTHERN CALIFORNIA | 6 |
| UNIVERSITY OF TEXAS HEALTH SCIENCE CENTER HOUSTON | 6 |
| VA MED CTR | 6 |
| YESHIVA UNIVERSITY | 6 |
| ALBANY MEDICAL COLLEGE | 5 |
| AUDIE L MURPHY MEMORIAL VETERANS HOSPITAL | 5 |
| DALHOUSIE UNIVERSITY | 5 |
| DEPAUL UNIVERSITY | 5 |
| HARVARD T H CHAN SCHOOL OF PUBLIC HEALTH | 5 |
| HENRY FORD HOSPITAL | 5 |
| JOHNS HOPKINS BLOOMBERG SCHOOL OF PUBLIC HEALTH | 5 |
| KAROLINSKA UNIVERSITY HOSPITAL | 5 |
| LOUISIANA STATE UNIVERSITY HEALTH SCIENCES CENTER NEW ORLEANS | 5 |
| NEVADA SYSTEM OF HIGHER EDUCATION NSHE | 5 |
| PENN STATE HEALTH | 5 |
| PHILIPPS UNIVERSITY MARBURG | 5 |
| RHODE ISLAND HOSPITAL | 5 |
| UNIVERSITY OF ARKANSAS SYSTEM | 5 |
| UNIVERSITY OF HOUSTON | 5 |
| UNIVERSITY OF HOUSTON SYSTEM | 5 |
| UNIVERSITY OF NEVADA RENO | 5 |
| UNIVERSITY OF NORTH CAROLINA SCHOOL OF MEDICINE | 5 |
| UNIVERSITY OF OTTAWA | 5 |
| UNIVERSITY OF TENNESSEE SYSTEM | 5 |
| UNIVERSITY OF TEXAS AUSTIN | 5 |
| VA PITTSBURGH HEALTHCARE SYSTEM | 5 |
| VET AFFAIRS PUGET SOUND HEALTH CARE SYSTEM | 5 |
| WALTER REED NATIONAL MILITARY MEDICAL CENTER | 5 |
| ALBERTA CHILDRENS HOSP RES INST | 4 |
| BOSTON MEDICAL CENTER | 4 |
| CHILDREN S HOSPITALS CLINICS OF MINNESOTA | 4 |
| CITY UNIVERSITY OF NEW YORK CUNY SYSTEM | 4 |
| DARTMOUTH COLLEGE | 4 |
| FLORIDA STATE UNIVERSITY | 4 |
| GHENT UNIVERSITY | 4 |
| INDIANA UNIVERSITY HEALTH | 4 |
| JESSE BROWN VA MEDICAL CENTER | 4 |
| JEWISH GENERAL HOSPITAL MONTREAL | 4 |
| LOUISIANA STATE UNIVERSITY HEALTH SCIENCES CENTER AT SHREVEPORT | 4 |
| MACQUARIE UNIVERSITY | 4 |
| MARY FREE BED REHABIL HOSP | 4 |
| MICHELI CTR SPORTS INJURY PREVENT | 4 |
| PALO ALTO UNIV | 4 |
| PENNSYLVANIA STATE UNIVERSITY UNIVERSITY PARK | 4 |
| RESEARCH TRIANGLE INSTITUTE | 4 |
| SWEDISH HEALTH SERVICES | 4 |
| SYRACUSE UNIVERSITY | 4 |
| UNITED STATES ARMY | 4 |
| UNITED STATES DEPARTMENT OF DEFENSE | 4 |
| UNIVERSIDAD DE MALAGA | 4 |
| UNIVERSITY HOSPITAL BRUSSELS | 4 |
